# Chimeric Antigen by the Fusion of SARS-CoV-2 Receptor Binding Domain with the Extracellular Domain of Human CD154: A Promising Improved Vaccine Candidate

**DOI:** 10.3390/vaccines10060897

**Published:** 2022-06-03

**Authors:** Ileanet Ávalos, Thailin Lao, Elsa María Rodríguez, Yasser Zamora, Alianet Rodríguez, Ailyn Ramón, Gilda Lemos, Ania Cabrales, Monica Bequet-Romero, Dionne Casillas, Ivan Andújar, Luis Ariel Espinosa, Luis Javier González, Yanitza Alvarez, Yamila Carpio, Mario Pablo Estrada

**Affiliations:** Center for Genetic Engineering and Biotechnology, CIGB, Ave. 31 E/158 y 190, Havana 10600, Cuba; ileanet.avalos@cigb.edu.cu (I.Á.); thailin.lao@cigb.edu.cu (T.L.); elsa.rodriguez@cigb.edu.cu (E.M.R.); yasser.zamora@cigb.edu.cu (Y.Z.); alianet.rodriguez@cigb.edu.cu (A.R.); ailyn.ramon@cigb.edu.cn (A.R.); gilda.lemos@cigb.edu.cu (G.L.); ania.cabrales@cigb.edu.cu (A.C.); monica.bequet@cigb.edu.cu (M.B.-R.); dionne.casillas@cigb.edu.cu (D.C.); ivan.andujar@cigb.edu.cu (I.A.); luis.espinosa@cigb.edu.cu (L.A.E.); luis.javier@cigb.edu.cu (L.J.G.); yanitza.alvarez@cigb.edu.cu (Y.A.)

**Keywords:** SARS-CoV-2, RBD, HEK-293, lentivirus, vaccine

## Abstract

COVID-19 is a respiratory viral disease caused by a new coronavirus called SARS-CoV-2. This disease has spread rapidly worldwide with a high rate of morbidity and mortality. The receptor-binding domain (RBD) of protein spike (S) mediates the attachment of the virus to the host’s cellular receptor. The RBD domain constitutes a very attractive target for subunit vaccine development due to its ability to induce a neutralizing antibody response against the virus. With the aim of boosting the immunogenicity of RBD, it was fused to the extracellular domain of CD154, an immune system modulator molecule. To obtain the chimeric protein, stable transduction of HEK-293 was carried out with recombinant lentivirus and polyclonal populations and cell clones were obtained. RBD-CD was purified from culture supernatant and further characterized by several techniques. RBD-CD immunogenicity evaluated in mice and non-human primates (NHP) indicated that recombinant protein was able to induce a specific and high IgG response after two doses. NHP sera also neutralize SARS-CoV-2 infection of Vero E6 cells. RBD-CD could improve the current vaccines against COVID-19, based in the enhancement of the host humoral and cellular response. Further experiments are necessary to confirm the utility of RBD-CD as a prophylactic vaccine and/or booster purpose.

## 1. Introduction

The coronavirus disease (COVID-19) is a respiratory viral disease caused by a new coronavirus called Severe Acute Respiratory Syndrome Coronavirus 2 (SARS-CoV-2). This virus has become a severe threat to global health due to its rapid expansion around the world with a high rate of morbidity and mortality [1]. SARS-CoV-2 belongs to the beta-coronavirus genus of the family *Coronaviridae.* The *Coronaviridae* family comprises 4 genera: *alpha-coronavirus*, *beta-coronavirus*, *gamma-coronavirus*, and *delta-coronavirus*. Coronaviruses are enveloped viruses with a single-stranded, positive-sense RNA genome of 29–30 kb in size. These viruses infect several animal species including humans [2]. The SARS-CoV-2 genome encodes four major structural proteins: spike (S), membrane (M), envelope (E), and nucleocapsid (N). The S protein has become the main therapeutic target due to its critical role in viral attachment, entry, and fusion. This protein has two major subunits: the S1 and S2. The S1 subunit contains a receptor-binding domain (RBD) of 25 kDa approximately, which is responsible for the direct binding with the angiotensin-converting enzyme 2 (ACE2), a protein receptor on the surface of human cells that mediates the entrance of the virus. The ACE2 receptor is expressed in specific subsets of human respiratory epithelial cells in nasal passages, airways, and alveoli. The RBD domain also causes the conformational changes of S2, resulting in virus fusion with and entry into the host cell for replication [3,4,5].

The use of S protein as immunogen was supported by previous evidences from SARS and MERS vaccine development, which indicate that strong antibody responses induced against the S protein have a significant effect on blocking viral entry into host cells during viral infection. Most of the SARS-CoV-2 neutralizing antibodies from COVID-19 convalescent patients were against the S protein or its RBD domain [6,7]. Additionally, vaccination with RBD has protected non-human primates (NHP) against the SARS-CoV-2 challenge, suggesting its potential to be further developed an effective preventive COVID-19 vaccine [8].

Multiple SARS-CoV-2 vaccines have been developed around the world using different platforms like mRNA, adenovirus vector, inactivated vaccines, and protein-based subunit vaccines [9,10,11,12,13,14]. However, despite the rapid production and distribution of vaccines, several new variants originating from various regions globally are constantly discovered. In addition, the necessary number of doses to achieve enough global coverage will not be possible with only one vaccine type. Also, long-term efficacy and safety data of these vector or mRNA vaccine platforms with a limited history of use, particularly in vulnerable individuals, are not available. In this scenario, a new generation of COVID-19 vaccines is needed to counteract the development of the pandemic [15,16,17]. Of especial interest are those vaccines that prevent viral infection in early post-immunization times with the fewest possible doses and that provide long-term protection by activating the memory T cell response [18,19,20].

The antigen design and the time needed for the production of large numbers of doses are the main constraints for the development of subunit vaccines as a first approach for emerging epidemic diseases. However, licensed subunit vaccines have proven tolerability and safety in many populations [21]. Several adjuvanted SARS-CoV-2 spike protein vaccines elicited neutralizing antibodies to protective levels in relevant animal models [22,23,24]. These advantages make subunit vaccines very attractive for controlling the long-term circulation of SARS-CoV-2, particularly if the virus continues provoking seasonal epidemic outbreaks of COVID-19.

In the present work, a new vaccine candidate against SARS-CoV-2 was developed. This vaccine candidate is based on the SARS-CoV-2 RBD protein domain fused to the extracellular domain of the human CD154 protein (RBD-CD). The CD154 is found on antigen-presenting cells, including B cells and in the surface of T cells. Several studies have claimed its potential as a molecular adjuvant, activating humoral and cellular immune responses [25,26,27,28]. The signals triggered by the binding of this molecule to its receptor (CD154-CD40) are essential for the proliferation and differentiation of the antigen-specific B cells and switching of antibody isotype and affinity maturation. The immune response that was activated by the CD154 signaling pathway is essential for the efficient generation of memory B cells and long-lived plasma cells [29,30]. The chimeric fusion protein was stably produced in HEK-293 cells using a lentivirus system. We discovered RBD-CD immunoreaction with sera from COVID-infected and recovered patients. The chimeric protein induced IgG-specific antibodies in mice and NHP after 2 doses of RBD-CD. The NHP sera inhibited the RBD binding to the ACE2 receptor in vitro and it also neutralized SARS-CoV-2 infection of Vero E6 cells. These data highlight the potential of RBD-CD vaccine candidate to develop as an effective vaccine against COVID-19.

## 2. Materials and Methods

### 2.1. Plasmids

To construct the plasmid pDisplay-CMV-RBD-CD, a linker sequence was first cloned into the pDisplay vector (Invitrogen, USA) downstream of the hCMV promoter/enhancer, and the plasmid pDisplay-CMV-linker was obtained. The linker was made by hybridization of synthetic oligonucleotides 1.1, 1.2, 2.1, and 2.2 (Table S1) that contain the nucleotide sequence corresponding to 6 His tail and a spacer (Ser-Gly-Gly-Gly-Ser-Gly-Gly-Gly-Gly-Ser-Gly-Gly-Gly-Gly-Ser), respectively. These oligonucleotides made it possible to incorporate the restriction sites for their subsequent cloning. Briefly, 50 pM of each oligonucleotide was hybridized. The hybridization reaction was performed at 94 °C for 2 min in such a way that oligo 1.1 was hybridized with oligo 1.2 (resulting in segment 1) and oligo 2.1 with oligo 2.2 (resulting in segment 2). Then, it was allowed to cool to room temperature and the concentration of each segment was quantified using a NanoPhotometer (IMPLEN, USA). The segments generated by the hybridization reactions were inserted using the T4 DNA ligase enzyme into the pDisplay vector previously digested with the restriction enzymes SacII-SmaI. Recombinant pDisplay-CMV-linker plasmids were detected by PCR, enzymatic digestion, and sequencing. In parallel, a sequence coding for 633 bp of the extracellular domain of Homo sapiens CD40 ligand (CD154) (NM_000074.2) was amplified by PCR from pCMV3-CD40LG-His (HG10239-CH, Sino Biological) using primers CD154-F and CD154-R (Table S1). The PCR product was digested with SacII and SalI and ligated downstream of the linker sequence in the plasmid pDisplay-CMV-linker digested with the same restriction enzymes, obtaining the plasmid pDisplay-CMV-linker-CD.

A sequence coding for residues 324–533 bp of the S protein of SARS-CoV-2 corresponding to the RBD domain was amplified by RT-PCR from mRNA samples purified from convalescent patients. Forward (RBD-F) and reverse primers (RBD-R) (Table S1) were designed based on the sequence reported for SARS-CoV-2/human/CHN/KMS1/2020 isolate (MT226610). The PCR product was cloned into a pGEM-Teasy vector (Promega, Madison, WI, USA) and sequenced. To clone the RBD into the plasmid pDisplay-CMV-linker-CD, the recombinant pGEM-Teasy vector clone was digested with BglII and SmaI and ligated upstream of the linker sequence in the plasmid pDisplay-CMV-linker-CD digested with the same restriction enzymes, obtaining the plasmid pDisplay-CMV-RBD-CD.

A third generation HIV-1-based lentivirus (LV) packaging system (Invitrogen, Waltham, MA, USA) was used to obtain lentiviral particles. The complete expression cassette (hCMV promoter/enhancer +Igk-chain leader sequence + RBD+ linker + CD154 extracellular domain) was amplified by PCR, adding a PstI restriction site to the 5′ end and a SalI restriction site to the 3′. The pLWRBD-CD-F and pLWRBD-CD-R oligonucleotide primers used are presented in Table S1. This PCR product was digested and ligated in the plasmid pLW digested with the same restriction enzymes, obtaining the plasmid pLW-CMV-RBD-CD.

### 2.2. Biological Reagents

The RBD-H6-HEK protein obtained in the culture supernatant of transiently transformed HEK-293T cells [Center for Molecular Immunology (CIM), Havana, Cuba] was used. This protein was obtained by metal chelate affinity chromatography purification and the final buffer was changed to PBS. The proteins hFc-RBD, hFc-ACE2, and mFc-ACE2 were also supplied by CIM, Havana, Cuba. In the case of hFc-RBD, it was conjugated with peroxidase (HRP) and was named hFcRBD-HRP [31]. Human, recombinant, Fc fusion protein (hFc-CD40) was purchased from BPS Bioscience (Catalog #71174). CIGB-300 [Center for Genetic Engineering and Biotechnology, (CIGB) Havana, Cuba] was dissolved as a 10 mM stock in PBS at room temperature for 5 min. For each experiment, a freshly-made stock was used. The drugs were diluted directly into growth media immediately previous to use.

### 2.3. Mammalian Cell Lines and Cell Culture Conditions

The original human embryonic kidney cells (HEK-293, ATCC CRL-1573) and FT variant cells (HEK-293FT, ATCC PTA-5077) were cultured in Dulbecco’s Modified Eagles Medium (DMEM, Sigma-Aldrich, St. Louis, MI, USA) with 10% fetal bovine serum (FBS, Capricorn) (DMEM-FBS) at 37 °C in 5% CO_2_. HEK-293FT cells were used as a packing cell line for the production of LVs and HEK-293 cells were used for lentiviral transduction and recombinant protein expression. The NCI-H460 cell line was obtained from the American Type Culture Collection (ATCC) and maintained in DMEM (Gibco, Thermo Fisher Scientific, Waltham, MA, USA) supplemented with 10% fetal bovine serum (Gibco, Thermo Fisher Scientific, Waltham, Massachusetts, USA), glutamine (300 μg/mL) (Gibco, Thermo Fisher Scientific, Waltham, Massachusetts, USA), and penicillin-streptomycin (100 U/mL) (Gibco, Thermo Fisher Scientific, Waltham, Massachusetts, USA).

The DMEM culture medium supplemented or not supplemented with FBS was also used for cell transduction, cloning procedures, and production of recombinant protein. The drug blasticidin (Invivogene, Waltham, MA, USA) was used as a selection agent at a final concentration of 6 µg/mL.

### 2.4. Plasmid Transient Expression

HEK-293 cells were seeded at 0.3 × 10^6^ cells per well in a six-well plate in 1 mL of DMEM-FBS and incubated at 37 °C in 5% CO_2_. Sixteen hours later, the metabolized culture medium was removed and the cell monolayer was gently washed three times with phosphate-buffered saline (PBS), pH 7.2 (Gibco, Thermo Fisher Scientific, Waltham, Massachusetts, USA), and 1.5 mL of DMEM culture medium was added per well. For transfection, 1 mg/mL linear polyethylenimine (PEI) (160 kDa) (Sigma-Aldrich, St. Louis, MI, USA) was used and the cells were transfected using a ratio of (1:1) (PEI: DNA). Ten micrograms of pDisplay-CMV-RBD-CD were used and 1 µg of a plasmid bearing green fluorescent protein (GFP) gene was also employed as a positive control. A mix of DNA, PEI, and 5% glucose was prepared and added directly to the cells. After 6 h of incubation at 37 °C in 5% CO_2_, 1 mL of FBS was added per well. Sixteen hours later, the serum-containing culture medium was removed and the cell monolayer was gently washed three times with PBS. Then, 3 mL of DMEM culture medium was added per well and cells were incubated at 37 °C in 5% CO_2_. The cell culture supernatant was harvested 72 h later. As a negative control of transfection, a mix of only PEI and 5% glucose was prepared and added to the cells, and transfection was performed following the same procedures as outlined above.

### 2.5. Production and Quantification of LVs

LVs were produced by transfection of HEK-293FT cells using linear PEI (160 kDa) and the third-generation HIV-1-based LV packaging system (Invitrogen), as previously described [32]. The Lenti-X™ Concentrator kit (Clontech) was used to purify the LVs following the manufacturer’s instructions. The LV pellet was diluted in 200 μL of DMEM culture medium and stored at −80 °C. An ELISA for detection of HIV p24 capsid protein (DAVIH-Ag p24, LISIDA, Havana, Cuba) was used for titration of the concentrated LV stocks.

### 2.6. Transduction of HEK-293 Cells Using LVs and Generation of RBD-CD-Expressing Cell Pools

The day before transduction, 2 × 10^4^ HEK-293 cells were seeded per well in a 24-well plate using 1 mL of DMEM-FBS culture medium and incubated at 37 °C in 5% CO_2_. After cells were checked under the microscope and an adherent and homogeneous monolayer was observed, the metabolized culture medium was removed and cell monolayer was washed with 1 mL of PBS. Transduction was performed by incubating LVs with cells in a DMEM culture medium at a final volume of 500 µL. Cells that were not transduced but were cultured with a selection drug (blasticidin) and were used as a control of transduction and drug selection (negative control). Two different MOI values were used: 50 and 100. Six hours post-transduction, 500 µL of selection culture medium 2X (DMEM, 20% FBS, 12 µg/mL blasticidin) were added to a final concentration of 6 µg/mL blasticidin. The second round of transduction was performed in the same conditions as outlined above. Twenty hours after the last round of transduction, the metabolized culture medium was gently aspirated and replaced with 1mL of selection culture medium (DMEM-FBS, 6 µg/mL blasticidin) to the control and transduced cells. This medium exchange was repeated every 48–72 h for approximately 21 days after the first time selection drug was added.

### 2.7. Cloning by Limiting Dilution and Evaluation of RBD-CD Expression Levels in Cell Pools and Clones

The cell pool generated at MOI 100 and resistant to 6 µg/mL of blasticidin was cloned by limiting dilution in 96-well plates [33].

To evaluate the RBD-CD protein expression levels in the cell culture supernatant in static culture, recombinant HEK-293 cell pools (derived from MOI 50 and MOI 100) and individual clones obtained by limiting dilution were seeded in 24-well plates at 0.4 × 10^6^ cells/well in 1 mL of DMEM-FBS culture medium. The experiment was performed in triplicate. Plates were incubated at 37 °C in 5% CO_2_ and after 9–10 days, cell culture supernatants were harvested to quantify RBD-CD protein-expression levels by ELISA.

### 2.8. SDS-PAGE

SDS-PAGE was performed as described [34]. Samples of cell culture supernatant or purified RBD-CD previously precipitated with TCA and sodium deoxycholate or applied directly were diluted in loading buffer [bromophenol blue 0.25%, dithiothreitol (DTT) 0.5 M, glycerol 50%, sodium dodecyl sulfate (SDS) 10%, and Tris-HCl 0.25 M pH 6.8] in a final volume of 30 µL for non-reducing conditions. For reducing conditions, samples were prepared with reducing–loading buffer (loading buffer + 25% 2-mercaptoethanol) for 10 min at 95 °C. Samples were loaded in 12.5% SDS-PAGE and after running stained with Coomassie Brilliant Blue G-250.

### 2.9. Immunoblotting Analysis

After protein electrophoresis, proteins were transferred to a nitrocellulose membrane (GE Healthcare). Membranes were blocked overnight at 4 °C with 5% skim milk in washing buffer (PBS pH 7.5, Tween-20 0.05%). After the blocking step, the membrane was washed three times for 5 min with washing buffer.

The membrane was incubated for 1 h at room temperature in shaken conditions with a horse-radish peroxidase (HRP)-conjugated mouse anti-His monoclonal antibody (1:2000, Sigma-Aldrich) diluted in PBS. Then, the membrane was washed again and the reaction developed.

Sera from convalescent patients were also used. All individuals gave their written informed consent for the use of their serum. The previously blocked membrane was incubated for 2 h at room temperature in shaken conditions with sera from convalescent patients (1:250). Then, the membrane was washed again and incubated with HRP-conjugated anti-human IgG (1:10,000, Jackson InmunoResearch) diluted in blocking buffer. After a step of incubation for 1 h at room temperature in shaken conditions, the membrane was washed again and the reaction developed.

For visualizing the proteins of interest previously marked by the HRP-conjugated antibodies, two different substrates were used: ECL detection system (Amersham) and 3,3′ diaminobenzidine (DAB).

### 2.10. Evaluation of RBD-CD Protein in HEK-293 Cell Culture Supernatant by ELISA

The evaluations of RBD-CD protein levels in HEK-293 cell culture supernatant were performed by ELISA. High binding Costar 96-well plates (Sigma-Aldrich, St. Louis, MI, USA) were coated with 5 μg/mL of hFc-ACE2 in coating buffer (0.1 M Na_2_CO_3_/NaHCO_3_, pH 9.6). The plates were incubated at 4 °C for 16 h and then they were washed three times with washing buffer (PBS + 0.05% Tween 20). Blocking was performed for 1 h at 37 °C with blocking solution (3% skim milk in PBS + 0.05% Tween 20). The samples were applied to the plates and incubated at 37 °C for 2 h. Then, the plates were washed three times with washing buffer. Afterward, a horseradish peroxidase (HRP)-conjugated mouse anti-His monoclonal antibody (1:20,000, Sigma-Aldrich) diluted in blocking solution was added and the plates were incubated at 37 °C for 30 min. The plates were washed again and a mix of 3,3–5,5-tetramethylbenzidine (TMB) and H_2_O_2_ diluted in citrate-phosphate buffer (pH 4.2) was added as substrate. The reaction was stopped with 2M H_2_SO_4._ Optical density was measured at 450 nm on a microplate reader.

### 2.11. Production of RBD-CD Protein in Batch Culture

Recombinant HEK-293 cells were seeded at 0.3–0.5 × 10^6^ cells/mL in 175 cm^2^ T-flasks in 25 mL of DMEM-FBS. When cells reached 90–100% of cell confluence, the monolayer was gently washed three times with PBS and 25 mL of medium DMEM (without serum) were added. Cells were incubated at 37 °C in 5% CO_2_. After 10 days, the cell culture supernatant was harvested and stored at −20 °C for further purification.

### 2.12. Protein Purification

Cell culture supernatant containing the RBD-CD protein was thawed and centrifuged at 4000× *g* for 10 min at 4 °C. The clarified cell culture supernatant was filtered through 0.45 µm and 0.2 µm cellulose nitrate filters (Sartorius). Afterward, protein purification was performed by metal affinity chromatography (IMAC). Briefly, filtered supernatant (250 mL) was equilibrated to a final concentration of PBS plus 2 mM imidazole (equilibrium buffer) by diluting in the same volume of PBS, and pH was adjusted to 7.4. Equilibrated supernatant was loaded at 0.5 mL/min at 4 °C onto an XK 16/40 column (GE Healthcare) packed with Ni-NTA agarose matrix (Qiagen, Hilden, Germany) previously equilibrated with 5 column volumes (CV) of equilibrium buffer. Then, the column was washed with 10 CV of washing buffer (PBS plus 10 mM imidazole, pH 7.4). The elution was performed with 5 CV of elution buffer (PBS plus 250 mM imidazole, pH 7.4). A buffer exchange to PBS was performed after purification with a PD10 desalting column (GE Healthcare, Chicago, IL, USA). The purified protein concentration was determined by measuring the absorbance at 280 nm (IMPLEN, Westlake Village, CA, USA). The purity of recombinant protein was assayed by densitometry scanning of protein gels, considering total protein concentration employing a BIO-RAD GS-800 scanner and the software Quantity One, version 4.6.9.

### 2.13. ESI-MS/MS Analysis

#### 2.13.1. Buffer-Free Digestion

The equivalent volume to 100 µg of protein in PBS (pH 7.3) was taken and deglycosylated with PNGase F (Biolabs, Durham, NC, USA) (1 µL) in the presence of guanidinium chloride (GuCl) 0.5 M during 2 h at 37 °C. The deglycosylated sample was cooled to room temperature (22 °C) and reacted with N-ethylmaleimide (NEM) 5 mM for 30 min. Next, 10 volumes of cold ethanol (−20 °C) final 80% were added and it was stored for 3 h at −80 ± 5 °C. The sample was centrifuged at 10,000× *g* for 10 min and the supernatant was removed. A total of three washes were performed with 500 uL of cold ethanol (−20 °C) 96% in water (−20 °C), and finally, it was centrifuged at 10,000× *g* for 10 min. The supernatant was removed and the sample was dried in SpeedVac (Savant, Hyannis, MA, USA) for 5 min. The precipitate was dissolved in 50 µL of ACN 20% in water, vortexed for 1 min, and kept in an ultrasound bath (Kaijo Denki, Shanghai, China) for 10 min. Then, 1 µg of modified trypsin (Promega, USA) was added in water and incubated for 16 h at 37 °C. 4 µL of digestion was taken and mixed with 0.3 µL of formic acid 90%. The sample was applied to a borosilicate microcapillary coated with a conductive material (Thermo Scientific, USA) and analyzed by mass spectrometry.

#### 2.13.2. Mass Spectrometry Analysis

The mixture of tryptic peptides was centrifuged at 10,000× *g* for 10 min. Then, 4 µL of the sample was withdrawn, acidified with 0.3 µL of formic acid 90%, homogenized by vortexing, centrifuged for 2 min at 10,000× *g*, and loaded into borosilicate capillaries covered with a conductive material (New Objective). Mass spectra were acquired with a hybrid orthogonal QTof-2TM tandem mass spectrometer (Micromass, Manchester, UK) by direct injection of the sample. Capillary and cone voltages of 1200 and 35 V, respectively, were used.

The ESI-MS spectrum of the digest was obtained in the *m*/*z* range of 200–2000 Th. The multiply-charged precursor ions were fragmented using collision energies between 20 and 50 eV. The collision gas was argon. The spectra were acquired and processed in the MassLynx v4.1 program.

#### 2.13.3. Protein Identification

Protein identification was performed with the MASCOT program (Matrix Science, version 2.5). The MS/MS spectra deconvoluted by MaxEnt v 3.0 (Micromass, UK) and exported as DTA format were used to identify proteins in the sequence databases. The search was performed using the SARS-CoV-2 and UP5640_H_sapiens protein databases available from software developers (http://www.matrixscience.com, accessed on 10 March 2021). The deamidation of asparagine and glutamine, the partial oxidation of methionine to sulfoxide, and the cysteine modifications were considered. The protease used was trypsin and a maximum of one missed cleavage site was allowed. The permissible mass error for precursor ions and fragment ions were 0.6 Da and 0.3 Da, respectively. The expected *m*/*z* values for the tryptic peptides including those linked by disulfide bonds were calculated by using the MassLynx v4.1 software (Micromass, Manchester, UK).

### 2.14. Surface Plasmon Resonance Experimental Procedure

Surface plasmon resonance (SPR) experiments were performed in a BIACORE X sensor (GE Health-care). In order to characterize the interaction of RBD-CD chimeric protein expressed in mammalian HEK-293 cells, with the receptor mFc-ACE2 chimeric protein, the latter was immobilized by capture on a Protein A biosensor chip (GE Health-care), according to the manufacturer’s protocol. The running buffer was PBS, pH 7.2 (10 µL/min). The real-time response of the RBD-CD over the immobilized mFc-ACE2 at 25 °C was recorded by triplicate in a single-cycle mode experiment. RBD-H6-HEK protein was also evaluated as a control. The experiment was conducted allowing both association and dissociation for 120 s, within a concentration range from 15 to 1000 nM. After each cycle, the chip was regenerated using pH 2.0 glycine buffer. The equilibrium dissociation constant (binding affinity constant, KD) was estimated with the BIAevaluation^®^ software (GE Health-care), fitting the experimental data to the Langmuir 1:1 interaction model with drifting baseline. At least five curves were considered for calculations.

### 2.15. Evaluation of RBD-CD and RBD-H6-HEK Binding to Human CD40

The evaluation of RBD-CD and RBD-H6-HEK binding to human CD40 was performed by ELISA. High-binding 96-well plates (Costar, Washington, DC, USA) were coated with 5 μg/mL of hFc-CD40 in coating buffer (0.1 M Na_2_CO_3_/NaHCO_3_, pH 9.6). The plates were incubated at 4 °C for 16 h then washed three times with washing buffer (PBS + 0.05% Tween 20). Blocking was performed for 1 h at 37 °C with blocking solution (3% skim milk in PBS + 0.05% Tween 20). Afterward, three washes in washing buffer were performed and serial dilutions (1:2) of RBD-CD or RBD-H6-HEK in blocking solution (0.78–100 µg/mL) were applied in triplicates. Plates were incubated at 37 °C for 2 h. Then, the plates were washed three times with washing buffer. Afterward, a horseradish peroxidase (HRP)-conjugated mouse anti-His monoclonal antibody (1:20,000, Sigma-Aldrich) diluted in blocking solution was added and the plates were further incubated at 37 °C for 30 min. The plates were washed again and a mix of 3,3–5,5-tetramethylbenzidine (TMB) and H_2_O_2_ diluted in citrate-phosphate buffer (pH 4.2) was added as substrate. The reaction was stopped with 2M H_2_SO_4._ Optical density was measured at 450 nm on a microplate reader.

### 2.16. In Vitro Proliferation Assay in NCI-H460 Cells

Cell proliferation was determined by crystal violet staining. Briefly, 20,000 H460 cells per well were seeded in flat-bottomed 96-well plates in DMEM medium with 10% FBS and incubated overnight at 37 °C at 5% CO_2_. Then, serial dilutions (1:10) of RBD-CD and RBD-H6-HEK (0.01–100 µg/mL) were added in triplicate. The antitumoral drug CIGB-300 was employed as positive control (6.25–100 µg/mL). After 72 h, the medium was removed from each well and crystal violet (1%) (Sigma-Aldrich, USA) was added and incubated for 5 min at room temperature. After dye removal, wells were washed with water. Finally, absorbance at 578 nm was read using a CLARIOstar^®^ high-performance monochromator multimode microplate reader (BMG LABTECH). The cell proliferation percentage was calculated using the following formula: cell proliferation (%) = ((OD of treated cells)/(OD of cell control)) × 100.

### 2.17. RBD-CD and RBD-H6-HEK Immunoreaction by Samples from Convalescent Patients

High binding 96-well plates (Costar) were coated with 10 μg of RBD-H6-HEK or 5 μg of RBD-CD both in coating buffer (0.1 M Na_2_CO_3_/NaHCO_3_, pH 9.6). After incubation at 4 °C for 16 h, the plates were washed four times with washing buffer (PBS + Tween 0.05%) and blocked for 1 h at 37 °C with blocking buffer (3% skim milk in washing buffer). Sera from naturally infected and recovered individuals were serially diluted 1:2 in blocking buffer and applied to the plates. After a step of incubation at 37 °C for 2 h, the plates were washed four times with washing buffer. Bound antibodies were detected with HRP-conjugated anti-human IgG (1:80,000, Jackson InmunoResearch, USA) diluted in blocking buffer. After another step of incubation at 37 °C for 1 h, the plates were washed again and a mix of TMB and H_2_O_2_ diluted in citrate-phosphate buffer (pH 4.2) was added as substrate. The reaction was stopped with 2M H_2_SO_4_ and absorbance was measured at 450 nm on a microplate reader. Samples were analyzed in duplicate. Sera from non-infected individuals were used as a negative control.

### 2.18. Animals and Immunization Schedules

#### 2.18.1. Immunogenicity in Mice

The immunogenicity of the chimeric RBD-CD was evaluated in 8-week-old female Balb/C mice weighing 15–18 g (CICUAL/CIGB/20099). The mice were divided into two experimental groups of nine mice each, one group immunized with 10 µg of RBD-CD and a negative control that was immunized with PBS. In all cases, 100 µL of the corresponding preparation was administered intramuscularly. Alhydrogel (Brenntag Biosector, Ballerup, Denmark) was used as an adjuvant at a final concentration of 1.44 mg/mL. The formulation was done for 16 h at 4 °C with shaking. The immunizations were performed on days 0 and 21. Retro-orbital blood samples were obtained at the beginning (pre-immune serum) and 21 and 35 days after beginning the experiment to evaluate specific IgG titers by ELISA. The collected blood was incubated at 4 °C for 16 h. Subsequently, it was centrifuged at 10,000× *g* for 20 min at 4 °C. The fraction corresponding to the serum was separated and stored at −20 °C until use.

#### 2.18.2. Comparison of Immunogenicy of RBD-CD and RBD-H6-HEK in Mice

Comparison between RBD-CD and RBD-H6-HEK in mice was performed in 8-week-old female Balb/C mice weighing 15–18 g (CICUAL/CIGB/21096). The mice were divided into three experimental groups of ten mice each: one group immunized with 10 µg of RBD-CD, one group immunized with RBD-H6-HEK, and a negative control that was immunized with PBS. In all cases, 100 µL of the corresponding preparation was administered intramuscularly. Alhydrogel (Brenntag Biosector, Denmark) was used as an adjuvant at a final concentration of 1.44 mg/mL. The formulation was done for 16 h at 4 °C with shaking. The immunizations were performed on days 0 and 21. Retro-orbital blood samples were obtained at the beginning (pre-immune serum) and 28 and 35 days after starting the experiment to evaluate specific IgG titers by ELISA. The collected blood was incubated at 4 °C for 16 h. Subsequently, it was centrifuged at 10,000× *g* for 20 min at 4 °C. The fraction corresponding to the serum was separated and stored at −20 °C until use. To evaluate the reactivation of the cellular response, the mice received a booster at day 112 and 14 days later, 5 animals from each group were sacrificed and the spleens were extracted under aseptic conditions. The cells were washed twice with 1% fetal bovine sera with 100 U/mL Antibiotic/Antimycotic Solution (Sigma-Aldrich, USA) in PBS (Sigma-Aldrich, USA). The cells (2 × 10^5^ cells per well) were seeded in 96-well round-bottom tissue culture plates in RPMI-1640 medium (Gibco, USA), supplemented with 100 U/mL Antibiotic Antimycotic Solution (Sigma-Aldrich, USA), 2 mM glutamine (Glutamax, Gibco, USA), and 5% FBS. The cells were stimulated for 72 h with RBD at 10 µg/mL and Concanavalin A (Sigma-Aldrich, USA) at 5 µg/mL was used as an assay positive control. The supernatants of splenocytes previously stimulated were analyzed by ELISA for mice (Mabtech, Germany), IL-2 (3311-1H-20), and IFNγ (3321-1H-20) following manufacturer’s instructions.

#### 2.18.3. Immunogenicity in Non-Human Primates

The immunogenicity of the chimeric RBD-CD protein was evaluated in *Macaca fascicularis* (male), from 3 to 4 years old, weighing from 2 to 4 kg (CICUAL/CIGB/21004). The monkeys were divided into two experimental groups of three monkeys each; one group corresponds to the negative control that was immunized with PBS while the other group was immunized with 50 µg of RBD-CD. Alhydrogel (Brenntag Biosector, Denmark) was used as an adjuvant at a final concentration of 1.44 mg/mL and was formulated for 16 h while moving at 4 °C. Two immunizations were performed intramuscularly in a volume of 0.5 mL. Immunizations were performed on days 0 and 21. Blood draws were performed after sedation with ketamine hydrochloride (10 mg/Kg) from the femoral vein at times 0 (pre-immune serum), 21, 28, 35, 42, and 63 to evaluate the humoral response. Serum was obtained and stored as previously described. The monkeys were monitored for body weight and rectal temperature throughout the experiment. Blood samples were collected on days 0, 42, and 228 to evaluate different hematological and biochemical parameters as a paid service, in the Center for Production of Laboratory Animals (CENPALAB, Bejucal, Cuba).

Specific anti-RBD titers and the inhibition of RBD interaction with the ACE2 receptor were evaluated using ELISA. Live SARSCoV-2 neutralization was assessed using a microneutralization assay.

### 2.19. Serum Antibodies Evaluation by ELISA

Antigen-specific IgG levels in the sera of the immunized animals (mice or NHP) were measured by ELISA. High binding 96-well plates (Costar, USA) were coated with 50 µL per well of a 10 μg/mL of RBD-H6-HEK or 5 μg/mL of RBD-CD solutions in coating buffer (0.1 M Na_2_CO_3_/NaHCO_3_, pH 9.6) for the evaluation of immunogenicity in mice. For the comparison of RBD-H6-HEK and RBD-CD in mice and for the study in NHP, RBD-H6-HEK was used as coating antigen as described above. After incubation at 4 °C for 16 h, the plates were washed four times with washing buffer (PBS + Tween 0.05%) and blocked for 1 h at 37 °C with blocking buffer (3% skim milk in washing buffer). Mouse or monkey sera from immunized mice were serially diluted in blocking buffer and applied to the plates. After a step of incubation at 37 °C for 2 h, the plates were washed four times with washing buffer. Bound antibodies were detected with HRP-conjugated goat anti-mouse IgG (1:10,000, Sigma-Aldrich) and HRP-conjugated anti-human IgG (1:80,000, Jackson InmunoResearch, USA) diluted in blocking buffer. After another step of incubation at 37 °C for 1 h, the plates were washed again and a mix of TMB and H_2_O_2_ diluted in citrate-phosphate buffer (pH 4.2) was added as substrate. The reaction was stopped with 2 M H_2_SO_4_ and absorbance was measured at 450 nm on a microplate reader. Samples were analyzed in duplicate. Antibody titer was defined as the reciprocal of the maximum dilution for which the absorbance values corresponding to serum dilutions were greater than twice the absorbance obtained for pre-immune serum.

### 2.20. RBD to ACE2 Plate-Based Binding Assay

ELISA plates were coated with 5 μg/mL of recombinant hFc-ACE2 in coating buffer in a volume of 50 μL for 16 h at 4 °C. A serum dilution of 1:100 was prepared in 0.2% skim milk in PBS-Tween 0.05% and a mixture containing an hFcRBD-HRP conjugate and diluted serum was pre-incubated for 1 h at 37 °C.

Overnight-coated plates were washed three times with PBS-Tween 0.05% and blocked with 2% skim milk in PBS-Tween 0.05% for 1 h at 37 °C. Afterward, the plates were washed three times and 50 µL of the mixture was added to hFc-ACE2-coated plates and further incubated for 60 min at 37 °C. The binding of the HRP tagged RBD to the receptor was detected after the addition of TMB and reading at 450 nm. The inhibition percentage was calculated as (%) = (1 − (OD_450nm_ mixtures/OD_450nm_ DOCN)) × 100, where the optical densities (OD) are the average of the absorbances of replicates.

The validity criteria for the assay was A450 of background <0.15 and negative control absorbance and maximum binding >0.6.

The sample with values ≥30% were considered to be positive for antibodies that inhibit the binding of RBD to ACE2.

### 2.21. Microneutralization of Live SARS-CoV-2 Virus in Vero E6 Cells

The neutralization antibody titers were detected by a traditional virus microneutralization assay using SARS-CoV-2 (CUT2010-2025/Cuba/2020 strain) according the procedure described by Limonta-Fernández, M., et al. [31]. The highest serum dilution indicating an OD value greater than the cut-off was considered as the neutralization titer. The cut-off value is calculated as the average of the OD of the cell control wells divided by two.

### 2.22. Statistical Analysis

Results were evaluated using GraphPad Prism version 8.0 for Windows (GraphPad Software). Treatments were considered to be significantly different if *p* < 0.05.

## 3. Results

### 3.1. Purification and Characterization of RBD-CD from Recombinant HEK-293 Culture Supernatant

**Figure 1:**
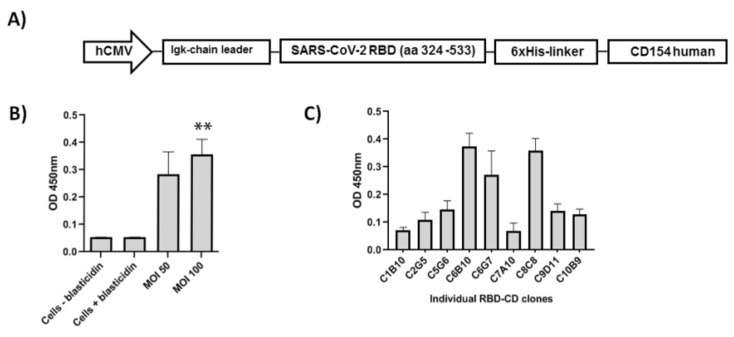
Construction and expression of RBD-CD in HEK-293 cells. (**A**) Schematic representation of the construction encoding the protein RBD-CD. The diagram indicates in that order from the *N*- towards the *C*-terminal end: a fragment of the human cytomegalovirus enhancer/promoter (hCMV), the Igk-chain leader sequence, a segment of RBD, a six-histidine tail plus a linker, and the extracellular domain of human CD154. (**B**) Protein expression in cell pools obtained after lentiviral transduction and selection with blasticidin determined by ACE2 binding in ELISA. Data are represented as mean + STD of six replicates. A Kruskal–Wallis and Dunn’s multiple comparison test were performed. Asterisks represent statistical differences with controls. (** *p* < 0.01) (**C**) ELISA to compare individual clones obtained by limiting dilution. Data are represented as mean + STD of three replicates.

The chimeric antigen has a modular structure where it is arranged from the *N-* to the *C-* terminal end as follows: (i) a segment of RBD (aa 324–533), (ii) a six histidine tail, (iii) a linker, and (iv) the extracellular domain of human CD154 (Figure 1A). Once confirmed transient expression by western blot (data not displayed), lentiviral particles were produced, quantified, and used for transduction of HEK-293 cells. Cells were selected employing blasticidin. Pools of resistant cells were obtained with both MOI employed. The protein expression in the culture supernatant obtained from the two different cell pools and individual cell clones was evaluated by binding to hFc-ACE2 in ELISA tests. The results indicated protein expression in culture supernatant from both pools and individual clones (Figure 1B,C). The results also demonstrated direct binding of RBD-CD to ACE2 receptor. The cell pool obtained employing MOI 100 was selected for further cloning due to highest expression detected in culture supernatant (Figure 1B). Among the three clones with the highest OD values in ELISA (C6B10, C6G7, and C8B8), clone C6B10 was selected to obtain recombinant RBD-CD for purification and further experiments. Future experimental work will achieve the adaptation of several clones to a protein-free and animal-derived component free in order to select the cell clone with the best growth performance and protein expression levels.

**Figure 2:**
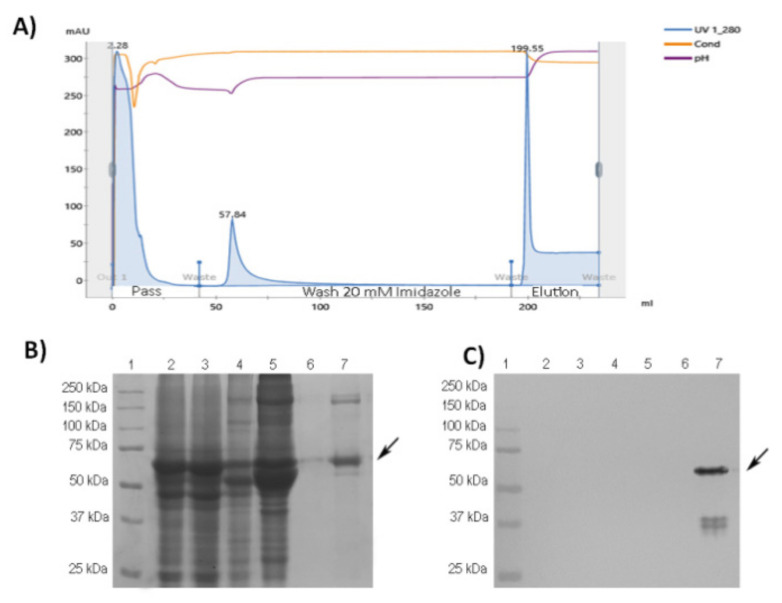
Purification of RBD-CD protein from the culture supernatant of stably transformed HEK293 cells. (**A**) Chromatogram of IMAC purification. Main purification steps are indicated by UV absorbance at 280 nm, pH, and conductivity; they comprise the flow-through after equilibrating the column, a washing step, and elution of purified RBD-CD. (**B**) Coomassie Blue stained 12% SDS-PAGE gel under reducing conditions. (**C**) Western blotting using anti-His tail antibody 1:2000. Lane 1: molecular weight marker, lane 2: initial sample, lane 3: material not bound to the matrix (column flow-through), lanes 4 and 5: 20 mM imidazole wash, lanes 6 and 7: elution at 250 mM imidazole. The arrow indicates the target protein.

The protein RBD-CD was purified from the serum-free supernatant by IMAC (Figure 2). The His-tag facilitated an efficient purification of the antigen, following steps of centrifugation for cell pellet separation, 0.2 µm filtration, and immobilization/elution in the Ni-NTA column. The protein indicated high affinity to the column since negligible RBD-CD amounts were detected in the column flow-through fraction after loading a maximum of 500 mL of diluted serum-free culture supernatant. For the optimization of this process, different imidazole concentrations were tested. Washing the column with 20 mM imidazole was found to be sufficient for the removal of most impurities, without appreciable losses of the RBD-CD. The RBD-CD was eluted at 250 mM imidazole concentration (Figure 2B,C, lane 7 corresponding to elution peak in the chromatogram). A band at the expected size according the predicted molecular weight of 51.4 kDa was detected with the anti-His monoclonal antibody. RBD-CD has 3 potential *N*-glycosylation sites (2 in RBD and 1 in CD154) but these modifications did not significantly increase protein molecular size according to SDS-PAGE and western blotting analysis. After purification, the RBD-CD was obtained with approximately 73% purity.

**Figure 3:**
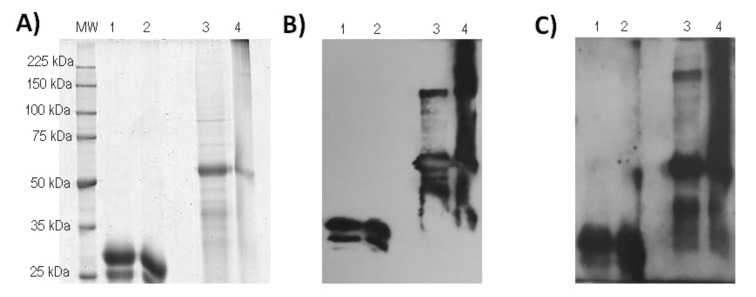
Comparison of the RBD-H6-HEK and RBD-CD proteins, both obtained in the same expression system, the HEK-293 cell line. (**A**) Coomassie Blue stained 10% SDS-PAGE gel under non-reducing conditions (NRC) and reducing conditions (RC). (**B**) Western blotting using an anti-His monoclonal antibody (dilution 1:2000) under NRC and RC. (**C**) Western blotting using sera from SARS-CoV2 naturally infected and recovered patients (dilution 1:250) and HRP-conjugated anti-human IgG (dilution 1:10,000), under NRC and RC. MW: molecular weight marker, lane 1: RBD-H6-HEK RC, lane 2: RBD-H6-HEK NRC, lane 3: RBD-CD RC, lane 4: RBD-CD NRC.

Figure 3 portrays SDS-PAGE and western blot analysis under reducing and non-reducing conditions of RBD-H6-HEK and RBD-CD proteins obtained in HEK-293 cell line. The RBD-H6-HEK and RBD-CD proteins migrate at the expected size of 27 kDa and 51.4 kDa respectively, under reducing conditions (Figure 3A). However, in non-reducing conditions, the RBD-CD band becomes faint and higher molecular weight aggregates are observed. These high molecular weight aggregates are not observed in the RBD-H6-HEK protein. Both proteins are recognized by the anti-His monoclonal antibody (Figure 3B) and by sera from SARS-CoV-2 naturally infected and recovered patients (Figure 3C). As ECL is a very sensitive detection technique, the presence of a possible dimer in the western blot under reducing conditions for RBD-CD (Figure 3B,C) could be due to incomplete reduction by mercaptoethanol.

### 3.2. ESI-MS Analysis of the Trypsin Digestion

**Figure 4:**
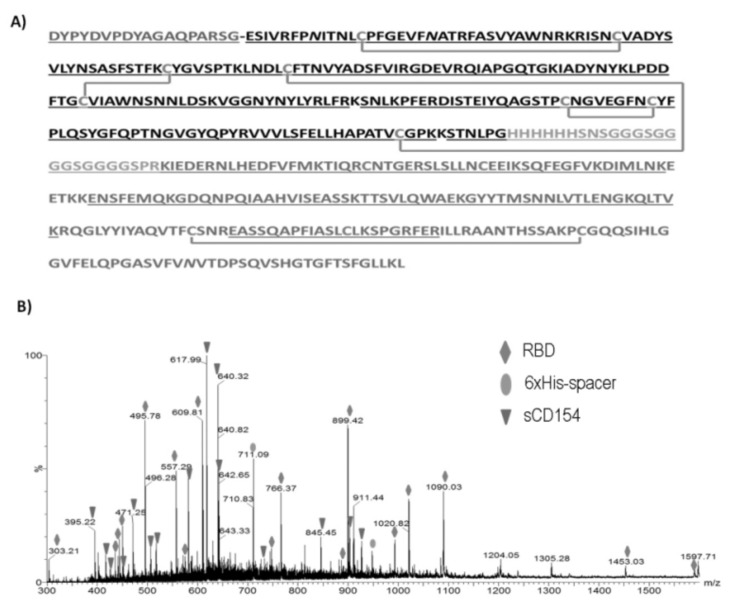
RBD-CD ESI-MS characterization (**A**) Amino acid sequence of the RBD-CD recombinant protein. The verified sequence by ESI-MS/MS analysis is underlined. The first residues correspond to additional 18 amino acids from the pDisplay subcloning vector. In black the receptor-binding domain (RBD) of SARS-CoV-2 S protein (residues Glu324-Gly535). The lines represent disulfide bonds that link C336-C361, C379-C432, C480-C488, and C391-C525 (for RBD) and C178-C218 for CD154. Residues indicated as *N* correspond to potential N-glycosylation sites. (**B**) ESI-MS spectrum of tryptic digestion of the RBD-CD protein in a buffer-free solution. The signals marked with diamonds correspond to the RBD protein peptides, the triangles to the CD154 fragment, and the ovals to the six His tails plus the spacer arm.

The RBD-CD protein (Figure 4A) was deglycosylated with PNGase F and digested with trypsin by a procedure known as in-solution buffer free digestion. The resultant peptides were directly analyzed by ESI-MS (Figure 4B) and their assignments are summarized in Table S2. Ninety-nine percent of the RBD sequence was verified including the presence of eight cysteine residues linked by four intra-molecular disulfide bonds (C^336^-C^361^, C^379^-C^432^, C^391^-C^525^, and C^480^-C^488^) identical to those present in the native RBD of SARS-CoV-2 (Table S2 and Figure 4A). The assignment of the peptides linked by disulfide bonds was also confirmed by ESI-MS/MS analysis (data not displayed). The signal detected at *m*/*z* 947.42, 3+ (see peptide T21 in Table S2) was assigned to the spacer arm between RBD and CD154 containing the six His residues in tandem used to purify the chimeric protein by IMAC. From all these results it was possible to verify the 82.36% of the RBD-CD protein. The ESI-MS analysis of the RBD-CD protein confirmed the presence of two out of the three potential N-glycosylation sites in the sequence. These results allowed to us unequivocally assert that the analyzed protein corresponds to the protein of interest and contains the RBD and CD154 domains.

### 3.3. Surface Plasmon Resonance Characterization of the RBD-ACE2 Binding

**Figure 5:**
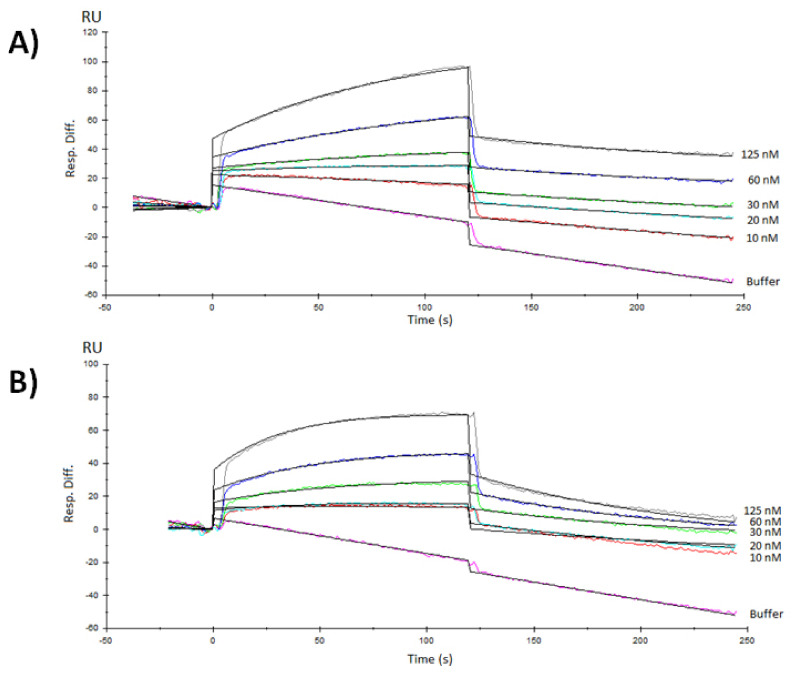
Surface plasmon resonance analysis from RBD recombinant proteins obtained in HEK-293 mammalian cells interacting with mFc-ACE2 cell receptor immobilized by capture in a Protein A sensor chip. (**A**) Sensorgrams of one of the replicates of the RBD-CD chimeric protein dissolved in PBS, pH 7.2. (**B**) Sensorgrams of one of the replicates of the RBD-H6-HEK tagged protein also dissolved in PBS, pH 7.2. Each color curve corresponds to the protein concentration indicated on the right edge. Black lines are the curves of the Langmuir 1:1 fitting, with drifting baseline (Chi^2^ ≤ 0.6).

As it is indicated in Figure 5, there was proportionality between response, with respect to resonance units (RU) and protein concentration for both RBD samples assayed, in contrast with the protein used as a negative control that did not display a detectable response (data not displayed). The association rate for RBD-CD protein, estimated through the experimental data fitting to the Langmuir 1:1 model with drifting baseline, was 6.9 ± 0.1 × 10^4^ M^−1^ · s^−1^, while the dissociation rate was 5.7 ± 0.7 × 10^−3^ s^−1^. A dissociation constant (KD) was 82.9 ± 11.7 × 10^−9^ M. The RBD-H6-HEK displayed an association rate of 1.6 ± 0.3 × 10^5^ M^−1^ · s^−1^ and a dissociation rate of 1.0 ± 0.1 × 10^−3^ · s^−1^, for a resulting KD = 67.2 ± 5.8 × 10^−9^ M.

### 3.4. CD40 Binding and Signaling in NCI-H460 Cells

**Figure 6:**
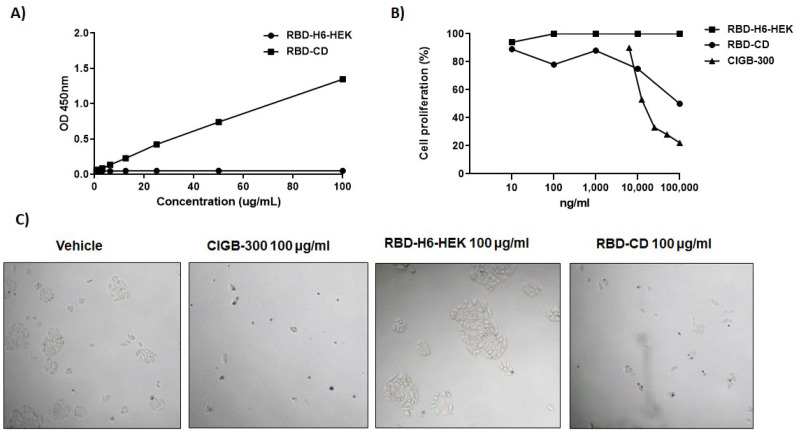
CD40 binding and signaling activation in vitro. (**A**) Binding ELISA using human Fc-CD40 as coating antigen. (**B**) Effect of RBD-CD on cell growth of NCI-H460 cells using crystal violet staining. The lung cancer cell line was cultured for 72 h with increasing concentrations of RBD-CD, RBD-H6-HEK, and CIGB-300. (**C**) Antiproliferative effect of RBD-CD in the lung cancer cells NCI-H460 using light microscopy (magnification 10×).

The binding of the extracellular domain of human CD154 within the chimera RBD-CD to its receptor CD40 was determined by ELISA using human Fc-CD40 as a coating antigen. The result indicated that the RBD-CD protein interacts with the CD40 receptor, presenting absorbance values above 1. The increase in the absorbance values was evidenced as RBD-CD protein concentration increased. However, absorbance values similar to blanks were observed for RBD-H6-HEK protein used as a negative control of the assay, as expected (Figure 6A). The effect of RBD-CD on the growth of large cell lung cancer NCI-H460 was assessed by crystal violet staining. Figure 6B presents the corresponding dose-response curve in the presence of RBD-CD and RBD-H6-HEK. The results evidence that RBD-CD significantly inhibited the proliferation of the CD40 positive cell line NCI-H460 with 50% of inhibition at 100 µg/mL, while no effect was demonstrated with RBD-H6-HEK, further confirming the specific effect of the CD154 (Figure 6C).

### 3.5. Antigenicity Analysis of RBD-CD

**Figure 7:**
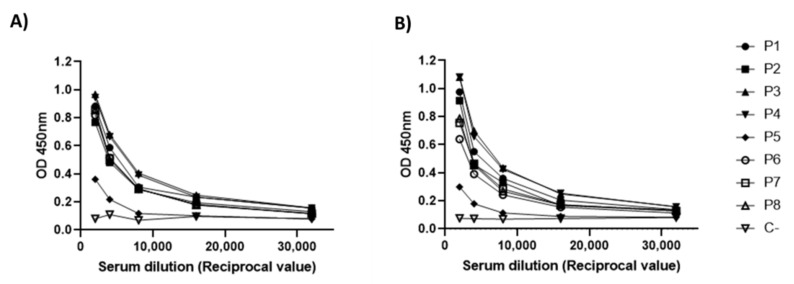
Immunoreaction of eight sera from patients naturally infected and recovered of COVID-19 (P1-P8) with the recombinant RBD-H6-HEK and RBD-CD. Serum was diluted from 1:2000 to 1:32,000. (**A**) ELISA using RBD-H6-HEK as coating antigen. (**B**) ELISA using RBD-CD as coating antigen. C-: Serum from a healthy donor.

To confirm the antigenicity of RBD-CD produced in HEK-293 cells, a panel of eight sera from patients naturally infected and recovered of COVID-19 was evaluated in an ELISA using either RBD-H6-HEK or RBD-CD as coating antigen. The sera used had neutralization titers for live SARS-CoV-2 on in vitro neutralization assays in Vero E6 cells. The results indicated that these sera have similar reactivity with both proteins, which suggests that immunogenic epitopes in the virus are well-conserved in recombinant RBD-CD. It also indicates that the fusion of CD154 extracellular domain to RBD does not significantly affect the structure of this viral protein in the structural context of the entire chimeric RBD-CD protein (Figure 7).

**Figure 8:**
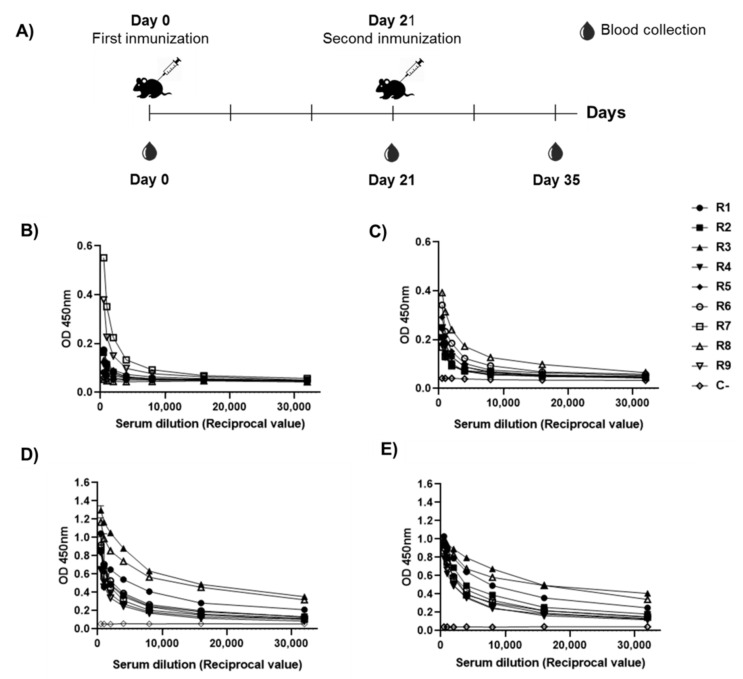
Immunogenicity studies in mice. (**A**) Schematic representation of immunization protocol and sample collection. The mice were divided into two experimental groups of nine mice each, one group immunized with 10 µg of RBD-CD and negative control that was immunized with PBS followed by a booster dose at 21 days after the first immunization. Mice sera were collected on day 0 (pre-immune serum) and at 21 and 35 days after starting the experiment to evaluate specific IgG titers by ELISA. Serum was diluted from 1:500 to 1:32,000. (**B**,**D**) ELISA using RBD-H6-HEK as coating antigen. (**C**,**E**) ELISA using RBD-CD as coating antigen. (**B**,**C**) 21 days after primary immunization. (**D**,**E**) 35 days after primary immunization (14 days post-booster). C-: Serum from the placebo group.

To evaluate the immunogenicity of RBD-CD protein, Balb/C mice were immunized with 10 μg of the protein, two doses at 21-day intervals, via the intramuscular route. ELISA tests were performed to evaluate specific IgG antibody response in sera, coating the plates either with RBD-H6-HEK or RBD-CD. As a result, a similar response was observed against both antigens, indicating that antibody response is directed primarily against RBD (Figure 8). Seroconversion of 100% of the animals was achieved two weeks after the boost. The administration of the booster at day 21 significantly increases RBD-specific IgG titers (Figure 8D,E).

### 3.6. Comparison between RBD-CD and RBD-H6-HEK in Mice

**Figure 9:**
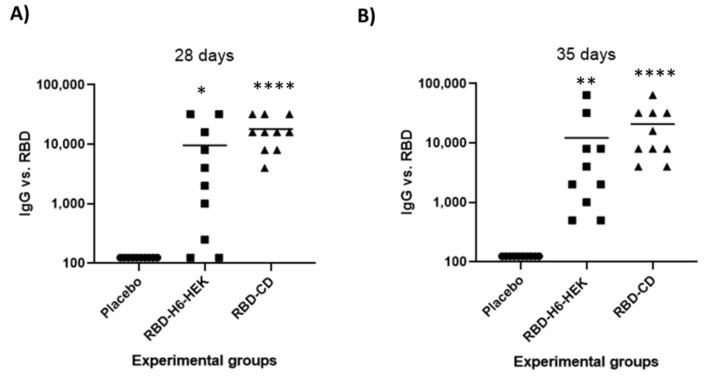
Comparison of immunogenicity between RBD-CD and RBD-H6-HEK in mice. The mice were divided into three experimental groups of ten mice each, one group immunized with 10 µg of RBD-CD, a second group immunized with RBD-H6-HEK, and a negative control that was immunized with PBS. Booster was done at 21 days after the first immunization. Mice sera were collected on day 0 (pre-immune serum) and at 28 (**A**) and 35 (**B**) days after starting the experiment to evaluate specific IgG titers by ELISA. Serum was diluted starting from 1:250. A Kruskal–Wallis and Dunn´s multiple comparison tests were performed. Asterisks represent statistically significant differences (*) *p* < 0.05, (**) *p* < 0.01, (****) *p* < 0.0001.

To compare the immunogenicity of RBD-CD protein with RBD-H6-HEK, Balb/C mice were immunized with 10 μg of the protein, two doses at 21-day intervals, by the intramuscular route. The results from the ELISA test to evaluate IgG antibody response at day 28 indicated that both proteins induced significant specific IgG titers compared to the placebo group, but the response induced by RBD-CD was higher (*p* < 0.0001) compared to the group immunized with RBD-H6-HEK (*p* = 0.0175) (Figure 9A). Seroconversion of 100% of the animals in the RBD-CD group was achieved as early as one week after the boost compared to 80% in RBD-H6-HEK immunized group. Two weeks after boost, despite all animals in RBD-H6-HEK being seroconverted, differences in antibody titers were maintained with *p* values < 0.0001 for RBD-CD and *p* = 0.0035 for RBD-H6-HEK (Figure 9B) compared to the placebo group. As it is observed in Figure 9, the IgG response after immunization with RBD-CD was more homogeneous compared to RBD-H6-HEK.

**Figure 10:**
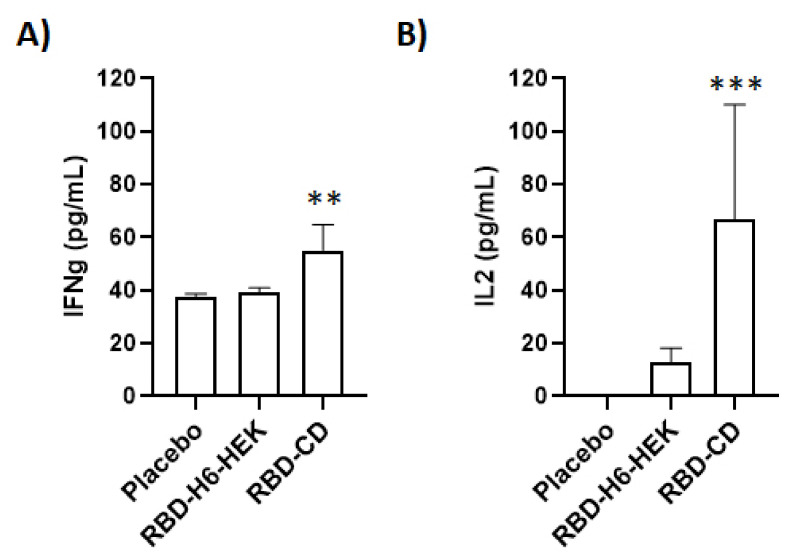
Comparison of immunogenicity between RBD-CD and RBD-H6-HEK in mice. To evaluate the reactivation of the cellular response, the mice received a booster at day 112 and 14 days later, 5 animals from each group were sacrificed and the spleens were extracted under aseptic conditions. The cells (2 × 10^5^ cells per well) were seeded in 96-well round-bottom tissue culture plates in RPMI-1640 medium. The cells were stimulated for 72 h with RBD-H6-HEK at 10 µg/mL. Concanavalin A (Sigma-Aldrich, USA) at 5 µg/mL was used as the assay positive control. The supernatants of splenocytes previously stimulated were analyzed by ELISA for mouse (**A**) IFNγ and (**B**) IL-2. (**) *p* < 0.01, (***) *p* < 0.001.

To evaluate the reactivation of the cellular response, IFNγ and IL2 were measured in the supernatant of splenocytes isolated at day 126 from mice immunized at day 112. As it was indicated in Figure 10, the secretion of IFNg and IL-2 was enhanced only in the RBD-CD-immunized group after 72 h of in vitro stimulation with RBD-H6-HEK.

### 3.7. Study in Non-Human Primates

**Figure 11:**
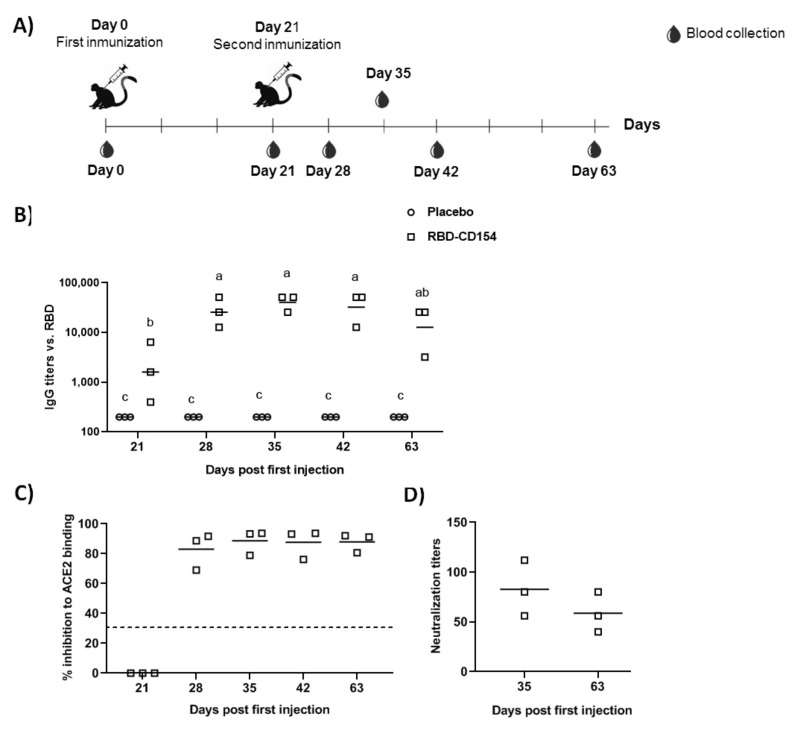
Experiment in non-human primates. (**A**) Schematic representation of immunization protocol and sample collection. The monkeys were divided into two experimental groups of three monkeys each; one group corresponds to the negative control that was immunized with PBS (placebo) while the other group was immunized with 50 µg of RBD-CD with alum adjuvant. Two immunizations were performed intramuscularly on days 0 and 21. Blood draws were performed at times 0 (pre-immune serum), 21, 28, 35, 42, and 63. (**B**) Evaluation of RBD-specific IgG response in immunized NHP. (**C**) Inhibition of ACE2 binding by a serum dilution 1:100. Samples with values ≥30% (discontinuous line) were positive for antibodies that inhibit the binding of RBD to ACE2. (**D**) Neutralization titers in a microneutralization assay of live SARS-CoV-2 virus in Vero E6 cells. Data are represented as individual plots and the median. A two-way ANOVA followed by Sidak´s multiple comparison test were performed after logarithmic transformation of titers. Different letters indicate statistically significant differences.

The RBD-CD protein evaluation in NHP using intramuscular administration schedule on days 0–21 and the dose of 50 µg indicates seroconversion of 100% after the first injection. The immune responses corroborated the boosting effect observed in mice. Average IgG titers increased from 2800 at 21 days to 29,867 and 42,667 at 28 and 35 days, respectively (Figure 11). The specific IgG antibody (Figure 10A) and the inhibition of RBD-ACE2 binding (Figure 11B) were only detected in RBD-CD protein-inoculated animals and not in control animals. The analysis of the neutralization titer of live SARS-CoV-2 virus in Vero E6 cells indicated that a 50 µg dose is sufficient to neutralize the virus at days 35 and 63. No binding inhibition titers or neutralizing activity was detected on day 21.

Throughout the study, the health of the monkeys was assessed by body temperature, weight, clinical signs, and hematological and biochemical parameters. The body temperature values of the animals were normal (Figure S1B). The monkeys did not demonstrate any significant weight loss (Figure S1C). Some hematological and biochemical parameters of the animals were measured before to start (day 0), after the first immunization (day 42), and at the end of experiment (day 228) (Tables S3–S5) and the results obtained did not indicate changes in the vaccinated group compared to the control group immunized with PBS.

## 4. Discussion

Despite the different types of vaccines against SARS-CoV-2 currently developed, research is still needed to develop more effective vaccines to mitigate the impact of the current pandemic and the appearance of new virus variants. This work focuses on the development and evaluation of a novel vaccine candidate against SARS-CoV-2 based on the RBD domain fused to the extracellular domain of CD154 through stable expression in the HEK-293 cell line. An RBD fragment covering amino acid residues 324 to 533 was selected to construct the chimeric RBD-CD protein. The RBD domain in SARS-CoV-2 comprises amino acid residues from 318 to 510 [35,36]. A previous study indicated that a recombinant RBD (aa 318–510) produced in CHO cells did not protect vaccinated mice from the SARS-CoV challenge, while a bigger fragment (aa 318–536) was able to induce complete protection [37]. The RBD segment selected for the RBD-CD construction contains 10 predicted CD4 T cell epitopes and 7 predicted CD8 T cell epitopes in addition to potential B cell epitope motifs, rendering a potentially effective immunogen according to previous reports [38,39]. To incorporate the CMV promoter and Igk-chain leader, the pDisplay vector was used for intermediate cloning based on the experience with other viral antigens and considering that strong gene expression and secretion of target protein to the culture medium is critical for further easy recovery and purification [40,41]. The SDS-PAGE results in non-reducing conditions indicated an aggregation phenomenon only observed for RBD-CD and not for RBD produced in the same expression system. This result suggests that this protein may be more immunogenic than RBD-H6-HEK [42,43].

The presence of eight cysteine residues linked by four intra-molecular disulfide bonds (C336-C361, C379-C432, C391-C525, and C480-C488) identical to those present in the native RBD of SARS-CoV-2 was confirmed by ESI-MS/MS analysis. The correct arrangement of disulfide bonds in the RBD is essential to obtain a correctly folded protein with the expected biological activity given by its ability to bind to the human ACE2 receptor and the production of neutralizing antibodies in the immunization of humans and animals [44].

The KD obtained for RBD-CD as result of the surface plasmon resonance characterization of the RBD-ACE2 binding was in the nanomolar range, as expected for this interaction pair, in compliance with previous reports [5,45,46,47]. The results confirmed that CD154 fusion at the C-terminal of RBD did not affect its binding to ACE2 receptor and that the RBD protein in the context of the chimeric protein is correctly folded. These results, along with other experimental pieces of evidence on these proteins’ antigenicity, suggest that the RBD obtained in mammalian cells could elicit an effective antibody response, capable to block virus entry and replication by blocking RBD-ACE2 interaction in a natural SARS-CoV-2 infection. The results also confirmed that CD154 fusion at the C-terminal of RBD did not affect its binding to the ACE2 receptor.

An ELISA was performed to evaluate the recognition of the CD154 within the chimeric protein by its CD40 receptor. The results indicated that RBD-CD is capable of binding to its cellular receptor CD40. There was no recognition of CD40 by RBD-H6-HEK protein as expected. This result suggests that RBD fused at the N-terminal of CD154 does not affect its interaction with the CD40 receptor and, therefore, the CD154 extracellular domain is correctly folded. Based on this evidence, it is expected that CD154 within the chimera can trigger the signaling mechanism after CD154-CD40 interaction. In this connection, it was demonstrated that RBD-CD significantly inhibited cell proliferation of the NCI-H460 cell line. A previous study reported that CD40 signaling induced growth inhibition of CD40-positive lung cancer cells [48]. CIGB-300, an anticancer peptide, was used as a positive control of the experiment since it is reported that it inhibits tumor cell proliferation in vitro [49,50]. No cell proliferation inhibition was observed with RBD-H6-HEK, as expected, due to absence of CD154 extracellular domain in this protein.

Intrinsic immunogenicity of the RBD is limited in mice after a single or two doses, which reflects the inefficient recruitment of high-quality T follicular helper cells in the primary response [51,52,53]. According to the results obtained, CD154 addition improved antibody and cellular response in this animal model. Previously, it was reported that recombinant adenovirus 5 (rAd5) expressing CD40-targeted S1 was capable of inducing significant levels of IgG and neutralizing antibodies specific to MERS-CoV in immunized mice. In that study, the incorporation of CD154 as molecular adjuvant substantially enhanced the immunogenicity of S1 [27]. Other authors also demonstrated the induction of strong antigen-specific T and B cell response with vaccines targeting diverse viral antigens to CD40 expressing antigen-presenting cells, confirming the advantage over the non-targeted antigens [17,54,55,56,57]. More recently, a vaccine employing RBD (aa 318–541) fused to the C-termini of the anti-human CD40 humanized antibody was used to generate an αCD40-RBD vaccine. The use of this vaccine demonstrated a significant improvement of immunity in convalescent macaques with a unique dose without the need for an additional adjuvant. The authors claimed that this kind of subunit vaccine targeting the antigen to CD40-expressing cells may serve as a safe and efficient boosting strategy for SARS-CoV-2 vaccines based on RBD and convalescent patients, suggesting an important outcome of this approach in people with specific vulnerabilities in addition to children [17]. Considering the demonstrated advantages of using new-generation subunit vaccines targeting the antigen to CD40-expressing cells, our design proposed the use of the CD40 ligand in place of the antibody, to target the RBD (antigen), which is the main novelty of the work. Several reports suggest the use of murine, porcine, or avian CD154 as a molecular adjuvant enhancing the immune response for subunit vaccines based on chimeric proteins [58,59,60]. However, to our knowledge, this configuration has never been proposed for a human protein-based vaccine. This type of vaccine should be easier to produce and purify with high yields in comparison to the one with the antibody as targeting molecule due to its smaller size and complexity.

There have been observed differences in vaccine antigen immunogenicity between small and large animal models [61], indicating the value of NHP models in clarifying the translational pathway of prototypic vaccines to humans. A previous study established a rhesus macaque model of COVID-19 infection using the Beta variant to evaluate the immune protective effect of the RBD targeted protein subunit vaccine and inactivated whole virus particle vaccine. Both vaccines used in the study were adjuvanted by aluminum. These candidates significantly reduced lung inflammation and decreased viral load in the respiratory tract in vaccinated macaques after challenged to the Beta variant (B.1.351) [62]. Another study reports a recombinant vaccine by fusing the SARS-CoV-2 RBD (aa 331–524) with the Fc fragment of human IgG1 expressed in mammalian CHO cells. The RBD-Fc fusion protein was more antigenic than the monomeric RBD protein in mice and NHP experiments. The use of this vaccine on macaques provided protection against SARS-CoV-2 challenge at day 56 after three doses. The RBD-Fc fusion protein-immunized macaques indicated much lower or no viral RNA copies compared to the control group immunized with PBS and very mild histopathological changes in a few lobes of the lung [63].

A similar chimeric design to the one used in this work was developed before in our research department to produce a vaccine against classical swine fever virus (CSFV) based on viral E2 glycoprotein fused to the extracellular domain of the swine CD154 protein. The E2-CD154 subunit vaccine obtained in HEK-293 cells was able to fully protect pigs from CSFV at 7 days post-vaccination with only one dose of 50 or 100 µg administered intramuscularly [30]. This vaccine protects pigs from CSFV challenge at least for 9 months using a two-dose scheme (0–21), demonstrating its value to induce long-term protection [64].

We immunized NHP and Balb/C mice with a human extracellular domain of CD154, which share 74.64% and 99.05% amino acid identity with murine and macaque sequences, respectively. A previous study also used the murine model to evaluate the effects of immunization with E2-CD154 (swine extracellular CD154) subunit vaccine adjuvated or not in Montanide ISA50V2 [65]. Murine and swine extracellular domains of CD154 share 72.73% of amino acid identity (Figure S2). The results indicated that neutralizing antibody titers were up to 8 times higher in mice immunized with E2CD154 emulsified in Montanide ISA50 V2 than those measured in the sera of mice immunized with E2-CD154 dissolved in saline one week after the booster. Moreover, the neutralizing antibody titers against E2His at day 35 were 10 times lower than the average titers observed for E2-CD154 emulsified in the same adjuvant. Furthermore, IFNγ levels measured in splenocyte culture supernatants were higher in mice immunized with E2-CD154, suggesting that CD154 should be responsible for the stimulation of this cytokine production [65]. These evidences support the utility of this animal model used to evaluate the immune response induced by RBD-CD as a first approach.

To evaluate the safety of the RBD-CD vaccine candidate, since CD154 is an endogenous molecule of the immune system, rectal temperature, body weight, and biochemical and hematological parameters were measured during the development of the experiment in monkeys. The body temperature values of the animals were stable between 37 and 39.5 °C, which is considered normal and within an acceptable range for cynomolgus macaques [66]. In addition, the body weight of the animals did not vary significantly during the study. The biochemical and hematological parameters of the control group immunized with PBS and the group immunized with RBD-CD were similar throughout the study and they were within the range reported for this species [67]. These results demonstrate that the monkeys were healthy since no evident pathological clinical signs were observed from the beginning of the experiment until 7 months after the first immunization. In a phase I human clinical trial, replication-defective adenovirus encoding ISF35 (Ad-ISF35), a recombinant CD154 transgene, was used ex vivo to transduce chronic lymphocytic leukemia (CLL) cells. This study demonstrated the safety and clinical activity of Ad-ISF35 transduced autologous leukemia cells for CLL patients [68]. In addition, no patient developed anti-ISF35 antibodies or liver function abnormalities [69]. Other vaccines in a phase I human clinical trial used an endogenous molecule of the immune system to promote an immune response against the SARS-CoV-2 virus without evidence of any immunopathology. The CORVax12 DNA vaccine approach combines the co-administration of a plasmid containing IL-12 with a DNA-encoding the SARS-CoV-2 spike or “S” glycoprotein to enhance immunogenicity [70]. Early preclinical data demonstrated the induction of higher IgG and neutralizing responses against the S protein and the RBD as result of co-administration. There was no evidence of toxicity, weight loss >10%, cachexia, labored breathing, or hunched posture in mice that were vaccinated with CORVax12 [71].

Overall, the results obtained suggest that the chimeric antigen RBD-CD could improve the current vaccines against COVID-19, by the enhancement of the host humoral and cellular response. Further experiments are necessary to confirm the utility of RBD-CD as a prophylactic vaccine and/or for booster purpose.

## 5. Conclusions

This work focuses on the development of a novel vaccine candidate against SARS-CoV-2 based on the RBD domain fused to CD154 through stable expression in the HEK-293 cell line. Obtaining the RBD protein that closely resembles the wild-type viral protein is crucial for recognition by the ACE2 receptor and for generating protective immunity. HEK-293 cells are a suitable cell line to obtain recombinant proteins with the appropriate conformation and post-translational modifications, which could be inappropriate or have unexpected implications for vaccine efficacy if produced in non-human cell lines or other platforms. The characterization of the RBD-CD obtained demonstrated the presence of the four intramolecular disulfide bonds identical to those present in the native SARS-CoV-2 RBD. BIACORE and ELISA results proved that RBD-CD can bind to ACE2 receptor. Additionally, CD154-CD40 interaction was demonstrated for RBD-CD chimeric protein. The results of comparison between RBD-CD and RBD-H6-HEK indicated an improved antibody response in animals injected with RBD-CD 1–2 weeks after booster, reflected by 100% seroconversion, higher specific IgG titers, and a more homogeneous response. Also, secretion of IFNg and IL2 were enhanced in this group in splenocytes stimulated in vitro with RBD antigen two weeks after a third dose. All these results, together with the ability of NHP sera to neutralize the live virus infection to Vero E6 cells only after two doses, point the potential of this approach to enhance the immunogenicity of subunit vaccines. Further evaluation trials that include challenges with the virus are necessary to corroborate that incorporation of CD154 as a molecular adjuvant could effectively enhance the immunogenicity of RBD based vaccines.

## Data Availability

The data presented in this study are available in the article and the Supplementary Materials.

## Supplementary Materials

The following supporting information can be downloaded at: https://www.mdpi.com/article/10.3390/vaccines10060897/s1, Table S1. List of primers; Table S2. Sequence verification by ESI-MS analysis of the deglycosylated RBD-CD protein digested with trypsin by in-solution buffer free digestion method; Table S3. Hematological and biochemical parameter values of NHP before the firstImmunization; Table S4. Hematological and biochemical parameter values of NHP 42 days before the first immunization; Table S5. Hematological and biochemical parameter values of NHP 228 days before the first immunization; Figure S1. Health monitoring in non-human primates. (A) Schematic representation of overall study design. NHPs were intramuscularly immunized with 50 µg of RBD-CD or PBS (Placebo) using alum as an adjuvant on 0 and 21 days. Blood collection were performed at times 0, 42 and 228 for hematological and biochemical tests. (B) Body temperature, (C) Weight measured at different days post first injection. The dashed lines mark the acceptable temperature range reported for cynomolgus macaques; Figure S2. Multiple sequence alignment of extracellular domain of CD154 from different species. (A) Clustal Omega 2.1 (https://www.ebi.ac.uk/Tools/msa/clustalo/) was used to align the protein sequences of mouse, swine, macaque fascicularis and human extracellular domain of CD154. (B) Percent identity matrix of the aligned sequences created by Clustal Omega 2.1. (*) Positions with a single, fully conserved residue. (:) Positions with conservation between amino acid groups of similar properties. (.) Positions with conservation between amino acid groups of weakly similar properties.
