# Supplementary material for: Chimeric Antigen by the Fusion of SARS-CoV-2 Receptor Binding Domain with the Extracellular Domain of Human CD154: A Promising Improved Vaccine Candidate
Source: Vaccines (Basel). 2022 Jun 3;10(6):897. doi: 10.3390/vaccines10060897 (PMC9228316; doi:10.3390/vaccines10060897)
Supplement: Supplementary file 1 [file vaccines-10-00897-s001.zip › vaccines-1662301-supplementary.pdf]

Supplementary Table S1. List of primers

Primer name	Primer sequence
Oligo 1.1	5′-GGGCATCATCACCATCACCATTCGAATTCTGGCGG-3′
Oligo 1.2	5′-GCCGCCGCCAGAATTCGAATGGTGATGGTGATGATGCCC-3′
Oligo 2.1	5′-CGGCTCCGGAGGGGGAGGGAGCGGCGGAGGGGGCTCCCCGC-3′
Oligo 2.2	5′-GGGGAGCCCCCTCCGCCGCTCCCTCCCCCTCCGGA-3′
CD154-F	5′-ATATATCCGCGGAAGATAGAAGATGAAAGGAATC-3′
CD154-R	5′-GTATATGTCGACCTAGAGCTTGAGTAAGCCAAAGG-3′
RBD-F	5′-AGATCTGGAGAATCTATTGTTAG-3′
RBD-R	5′-CCCGGGCAAATTAGTAGAC-3′
pLWRBD-CD-F	5′-ATATATCTGCAGGCGCGCGTTGACATTGATTATTG-3′
pLWRBD-CD-R	5′- GTATATGTCGACCTAGAGCTTGAGTAAGCCAAAGG -3′

Supplementary Table S2. Sequence verification by ESI-MS analysis of the deglycosylated RBD-CD protein digested with trypsin by in-solution buffer free digestion method

Protein	Residue [a)]	Z	Theor m/z	Obs m/z	Sequence [b)]
Subcloning vector + RBD	17-23	1+	747.40	747.39	SGESIVR
RBD	(24-41)—(53-73)	3+	1452.35	1452.37	FPDITNL**C**PFGEVFDATR<br>I<br>ISN**C**VADYSVLYNSASFSTFK
		4+	1089.51	1089.54	
RBD	42-50	1+	1113.55	1113.53	FASVYAWNR
		2+	557.28	557.29	
RBD	(74-81)—(120-139)	3+	1020.81	1020.82	**C**YGVSPTK<br>I<br>LPDDFTG**C**VIAWNSNNLDSK
		4+	765.86	765.88	
RBD	(82-98)—(205-223)	4+	992.52	992.53	LNDL**C**FTNVYADSFVIR<br>I<br>VVVLSFELLHAPATV**C**GPK
RBD	99-103	1+	575.28	575.30	GDEVR
RBD	104-112	2+	450.25	450.26	QIAPGQTGK
RBD	113-119	1+	886.43	886.45	IADYNYK
		2+	443.72	443.73	
RBD	140-149	1+	1218.59	1218.57	VGGNYNYLYR
		2+	609.80	609.81	
RBD	150-152	1+	435.27	435.28	LFR
RBD	154-161	2+	495.77	495.78	SNLKPFER
RBD	162-204	3+	1589.38	1589.40	DISTEIYQAGSTP**C**NGVEGFN**C**YFPLQSYGFQPTN<br>I___________I<br>GVGYQ PYR
6xHis-spacer arm	225-255	3+	947.42	947.45	STNLPGHHH HHSN GGGS GGGS GGGS R
		4+	710.82	710.83	
sCD154	257-261	1+	661.32	661.34	IEDER
sCD154	262-271	1+	1279.61	1279.61	NLHEDFVFMK
		2+	640.31	640.32	
		3+	427.21	427.22	
sCD154	272-275	1+	517.31	517.32	TIQR
sCD154	282-292	2+	687.35	687.36	SLSLLN**C**EEIK<br>I<br>**nem**
sCD154	293-300	1+	941.47	941.47	SQFEGFVK
		2+	471.24	471.25	
sCD154	301-306	1+	733.39	733.39	DIMLNK
sCD154	312-319	2+	506.72	506.74	ENSFEMQK
sCD154	320-337	2+	926.46	926.47	GDQNPQIAAHVISEASSK
		3+	617.98	617.99	
sCD154	338-347	1+	1162.61	1162.63	TTSVLQWAEK
		2+	581.81	581.82	
sCD154	348-363	2+	902.43	902.44	GYYTMSNNLVTLENG K
sCD154	364-368	1+	588.37	588.35	QLTVK
sCD154	386-400	2+	845.43	845.45	EASSQAPFIASL**C**LK

					**I**<br>**nem**
sCD154	401-404	1+	416.23	416.25	SPGR
sCD154	405-407	1+	451.23	451.25	FER
Assignment of peptides containing one missed cleavage site of of trypsin					
sCD154	51-52	1+	303.21	303.21	KR
sCD154	256-261	1+	789.41	789.41	KIEDER
		2+	395.21	395.22	

a) The numbering of amino acids in this table corresponds to the position of the chimeric protein RBD-CD.

b) **D** means potential *N*-glycosylation sites that were transformed into Asp residues by PNGase F. **nem** corresponds to residues of free cysteine residues in the protein that were alkylated by *N*-ethylmaleimide in the processing of the sample before treatment with PNGase F. Lines connecting cysteine residues indicate disulfide bonds.

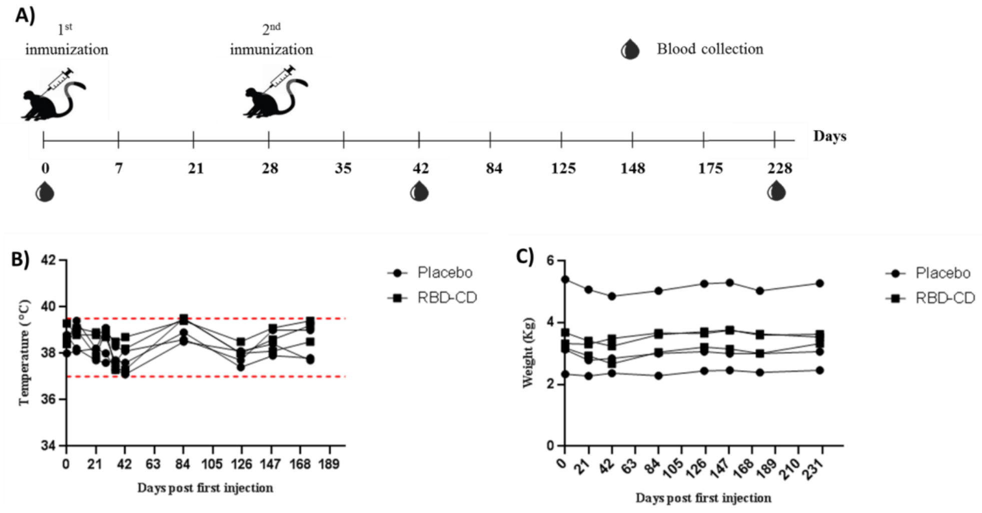

Supplementary Figure S1. Health monitoring in non-human primates. A) Schematic representation of overall study design. NHPs were intramuscularly immunized with 50 µg of RBD-CD or PBS (Placebo) using alum as an adjuvant on 0 and 21 days. Blood collection were performed at times 0, 42 and 228 for hematological and biochemical tests. B) Body temperature, C) Weight measured at different days post first injection. The dashed lines mark the acceptable temperature range reported for cynomolgus macaques.

Supplementary Table S3. Hematological and biochemical parameter values of NHP before the first immunization.

Parameters	Abbreviation	Unit	Placebo 1	Placebo 2	Placebo 3	RBD-CD154 1	RBD-CD154 2	RBD-CD154 3
Globulin index	A/G		1,5	1,7	1,3	1,6	2,2	1,7
Alanine amino transferase	ALAT	u/L	13	15,7	12,4	17,2	26	16
Aspartate amino transferase	ASAT	u/L	37	58	53	40	43	50
Alkaline phosphatase	ALP	u/L	305	540	351	305	394	337
Creatinine	CREA	µmol/L	85	55	42	53	46	72
Total proteins	TP	g/L	80	70,2	79,9	74,9	68,6	72,0
Albumin	ALB	g/L	51,2	43,4	45,8	49,4	47,4	45,0
Glucose	GLU	mmol/L	6,55	4,89	3,31	3,48	4,91	5,29
Cholesterol	CHOL	mmol/L	4,41	3,54	3,88	3,49	2,51	3,90
Total bilirubin	BIL-T	µmol/L	0	0,2	0	0	0	0,2
Direct bilirubin	BIL-D2	µmol/L	0,7	0,9	0	0,8	0,3	0,2
Triglycerides	TG	mmol/L	0,42	0,44	0,52	0,48	0,54	0,58
Phosphorus	PHOS	mmol/L	1,9	1,67	2,02	1,10	1,16	2,00
Urea	UREA	mmol/L	10,74	11,06	10,69	10,43	10,18	7,51
Calcium	Ca	mmol/L	2,56	2,49	2,42	2,57	2,57	2,46
Uric acid	UA	µmol/L	0	2	5	8	4	0
Gamma glutamyl transferase	GGT	u/L	80	132	69	62	66	64
Hemoglobin	HB	g/dL	14,6	13,5	11,8	13,9	12,7	14
Hematocrit	HTC	%	48,3	46,1	39,4	45,9	42,4	46,5
Erythrocyte	ETO	$10^3/mm^3$	1,91	7,06	6,6	7,63	6,96	7,58
Platelet	PLAT	$10^3/mm^3$	395	407	380	387	435	187
Medium corpuscular volume	MCV	fL	61	65	60	60	61	61
Mean corpuscular hemoglobin	MCH	pg	18,5	19,2	17,8	18,2	18,7	18,4
Mean corspuscular hemoglobin concentration	MCHC	g/dL	39,7	29,3	29,8	30,2	29,9	30,3

Supplementary Table S4. Hematological and biochemical parameter values of NHP 42 days before the first immunization

Parameters	Abbreviation	Unit	Placebo 1	Placebo 2	Placebo 3	RBD-CD154 1	RBD-CD154 2	RBD-CD154 3
Globulin index	A/G		1,5	1,4	1,6	1,7	1,5	1,3
Alanine amino transferase	ALAT	u/L	13	nd	13	35	26	16
Aspartate amino transferase	ASAT	u/L	40	34	43	41	44	39
Alkaline phosphatase	ALP	u/L	251	534	360	228	233	318
Creatinine	CREA	µmol/L	89	65	44	63	55	44
Total proteins	TP	g/L	82,9	70,9	76,7	82,8	86,1	71,9
Albumin	ALB	g/L	50,2	41,7	47,3	51,8	51,8	40,4
Glucose	GLU	mmol/L	6,21	4,34	3,84	5,17	5,31	5,26
Cholesterol	CHOL	mmol/L	4,16	3,35	4,2	3,92	4,94	2,87
Total bilirubin	BIL-T	µmol/L	2,4	2,5	1,6	5,3	2,3	1,4
Direct bilirubin	BIL-D2	µmol/L	0,9	1,2	1	2,0	1,0	0,6
Triglycerides	TG	mmol/L	0,26	0,83	0,66	0,44	0,63	0,54
Phosphorus	PHOS	mmol/L	2	2,18	1,96	1,85	2,04	1,99
Urea	UREA	mmol/L	10,31	8,71	10,01	9,99	8,87	9,71
Calcium	Ca	mmol/L	2,6	2,32	2,43	3,10	2,53	2,57
Uric acid	UA	µmol/L	0	1	1	0	0	0
Gamma glutamyl transferase	GGT	u/L	90	111	68	69	52	70
Hemoglobin	HB	g/dL	13	13,2	11,2	13,7	14,4	11,9
Hematocrit	HTC	%	45,9	46,9	40,9	48,8	41,8	41,6
Erythrocyte	ETO	$10^3/mm^3$	6,34	6,14	5,89	6,81	5,50	5,89
Platelet	PLAT	$10^3/mm^3$	665	378	403	408	514	405
Medium corpuscular volume	MCV	fL	72	76	69	72	76	71
Mean corpuscular hemoglobin	MCH	pg	20,5	21,5	19,1	20,1	26,2	20,3
Mean corspuscular hemoglobin concentration	MCHC	g/dL	28,3	28,1	27,5	28,1	34,4	28,6

Supplementary Table S5. Hematological and biochemical parameter values of NHP 228 days before the first immunization

**Parameters**	**Abbreviation**	**Unit**	**Placebo 1**	**Placebo 2**	**Placebo 3**	**RBD-CD154 1**	**RBD-CD154 2**	**RBD-CD154 3**
Globulin index	A/G		2,2	1,8	2,3	1,8	2,2	1,8
Alanine amino transferase	ALAT	u/L	17,4	13	10,1	11,8	13,7	11,2
Aspartate amino transferase	ASAT	u/L	24,4	36,9	33,8	68,2	27,0	35,9
Alkaline phosphatase	ALP	u/L	144	385	294	223	282	334
Creatinine	CREA	µmol/L	82	74	49	63	56	58
Total proteins	TP	g/L	77	74	77	71	68	70
Albumin	ALB	g/L	53	48	54	46	47	45
Glucose	GLU	mmol/L	6,13	5,3	3,3	4,58	4,20	3,67
Cholesterol	CHOL	mmol/L	2,89	3,23	4,22	2,89	3,63	3,29
Total bilirubin	BIL-T	µmol/L	1,2	1,4	1,2	2,4	2,3	0,7
Direct bilirubin	BIL-D2	µmol/L	0,7	0,6	0,6	0,8	0,8	0,5
Triglycerides	TG	mmol/L	0,5	0,71	0,62	0,48	0,89	0,99
Phosphorus	PHOS	mmol/L	1,71	1,71	2,02	1,92	1,62	1,26
Urea	UREA	mmol/L	13,5	11	14,3	8,7	8,9	10,9
Calcium	Ca	mmol/L	2,53	2,58	2,5	2,47	2,37	2,62
Uric acid	UA	µmol/L	1	1	1	4	1	0
Gamma glutamyl transferase	GGT	u/L	102	101	67	59	66	65
Hemoglobin	HB	g/dL	11,8	12,1	11,6	12,6	12,5	10,8
Hematocrit	HTC	%	41,2	43,3	40,8	44.8	43,0	39,0
Erythrocyte	ETO	$10^3/mm^3$	6,14	6,05	6,22	6,66	6,37	5,96
Platelet	PLAT	$10^3/mm^3$	373	285	428	587	583	378
Medium corpuscular volume	MCV	fL	67	72	66	67	68	65
Mean corpuscular hemoglobin	MCH	pg	19,2	20,0	18,6	18,9	19,6	18,5
Mean corspuscular hemoglobin concentration	MCHC	g/dL	28,5	27,9	28,4	28,1	29,0	27,7

```
CLUSTAL O(1.2.4) multiple sequence alignment
```

A)

```
CD40L_MOUSE      KVEEEVNLHEDFVFIKKLKRCNKGEGSLSLLNCEEMRRQFEDLVKDITLNKEE-KKENSF
CD40L_Swine      KIEDERNLHEDFVFIKTIQRCKQGEGSLSLLNCEEIRSQFEDLVKGIMQSKEVKKKEKSF
CD40L_HUMAN      KIEDERNLHEDFVFMKTIQRCNTGERSLSLLNCEEIKSQFEGFVKDIMLNKEETKKENSF
CD40L_MACFA      KIEDERNLHEDFVFMKTIQRCNTGEKSLSLLNCEEIKSQFEGFVKDIMLNKEEKKKENSF
                 *:*:* ********:*.::**: ** *********:: ***.:**.*  .**  ***:**

CD40L_MOUSE      EMQRGDEDPQIAAHVVSEANSNAASVLQWAKKGYYTMKSNLVMLENGKQLTVKREGLYYV
CD40L_Swine      EMHKGDQDPQIAAHVISEASSKTASVLQWAPKGYYTLSTNLVTLENGRQLAVKRQGIYYI
CD40L_HUMAN      EMQKGDQNPQIAAHVISEASSKTTSVLQWAEKGYYTMSNNLVTLENGKQLTVKRQGLYYI
CD40L_MACFA      EMQKGDQNPQIAAHVISEASSKTTSVLQWAEKGYYTMSNNLVTLENGKQLTVKRQGLYYI
                 **::**::*******:***.*:::****** *****:..*** ****:**:***:*:**:

CD40L_MOUSE      YTQVTFCSNREPSSQRPFIVGLWLKPSSGSERILLKAANTHSSSQLCEQQSVHLGGVFEL
CD40L_Swine      YAQVTFCSNRDAAGQAPFIASLCLRSPSGSERILLRAANTHSSSKPCGQQSIHLGGVFEL
CD40L_HUMAN      YAQVTFCSNREASSQAPFIASLCLKSPGRFERILLRAANTHSSAKPCGQQSIHLGGVFEL
CD40L_MACFA      YAQVTFCSNREASSQAPFIASLCLKSPGRFERILLRAANTHSSAKPCGQQSIHLGGVFEL
                 *:********: :.* ***..* *:  .  *****:*******:: * ***:********

CD40L_MOUSE      QAGASVFVNVTEASQVIHRVGFSSFGLLKL      209
CD40L_Swine      QPGASVFVNVTDPSQVSHGTGFTSFGLLKL      210
CD40L_HUMAN      QPGASVFVNVTDPSQVSHGTGFTSFGLLKL      210
CD40L_MACFA      QPGASVFVNVTDPSQVSHGTGFTSFGLLKL      210
                * *********: *** * .**:*******
```

B)

```
Percent Identity Matrix - created by Clustal 2.1

 1: CD40L_MOUSE   100.00    72.73    74.64    74.64
 2: CD40L_Swine    72.73   100.00    85.71    86.19
 3: CD40L_HUMAN    74.64    85.71   100.00    99.05
 4: CD40L_MACFA    74.64    86.19    99.05   100.00
```

Supplementary Figure S2. Multiple sequence alignment of extracellular domain of CD154 from different species. A) Clustal Omega 2.1 (https://www.ebi.ac.uk/Tools/msa/clustalo/) was used to align the protein sequences of mouse, swine, macaque fascicularis and human extracellular domain of CD154. B) Percent identity matrix of the aligned sequences created by Clustal Omega 2.1. (*) Positions with a single, fully conserved residue. (:) Positions with conservation between amino acid groups of similar properties. (.) Positions with conservation between amino acid groups of weakly similar properties.

Declaración de consentimiento informado

Yo, (Nombre y apellidos)

____

He recibido información acerca de la enfermedad conocida como Covid 19 y algunas investigaciones que se están realizando para encontrar vacunas efectivas contra la enfermedad.

He hablado con:

....
(Nombre del médico-investigador)

Accedo a que las muestras de sangre obtenidas puedan ser utilizadas para análisis relacionados con la enfermedad o vacunas en estudio.
Manifiesto mi conformidad y doy mi consentimiento para el acceso y utilización de mis datos.

Para constancia de lo expuesto anteriormente firmo este documento, en Ciudad de la Habana, el día _____________________________ del 2021.

________________________________ ________________________________

Firma del voluntario Firma del Investigador principal
